# Safety and efficacy of AK0529 in respiratory syncytial virus‐infected infant patients: A phase 2 proof‐of‐concept trial

**DOI:** 10.1111/irv.13176

**Published:** 2023-07-25

**Authors:** Li‐Min Huang, Andreas Schibler, Yi‐Chuan Huang, Andrew Tai, Hsin Chi, Chae‐Hee Chieng, Jinn‐Li Wang, Aviv Goldbart, Swee‐Ping Tang, Yhu‐Chering Huang, Shane George, Derya Alabaz, Lea Bentur, Siew‐Choo Su, Jessie de Bruyne, Bulent Karadag, Feng Gu, Gang Zou, Stephen Toovey, John P. DeVincenzo, Jim Z. Wu

**Affiliations:** ^1^ Department of Pediatrics, National Taiwan University Children's Hospital National Taiwan University Taipei Taiwan; ^2^ Pediatric Intensive Care Unit Queensland Children's Hospital South Brisbane Queensland Australia; ^3^ Department of Pediatrics Kaohsiung Chang Gung Memorial Hospital and Chang Gung University College of Medicine Kaohsiung Taiwan; ^4^ Department of Pediatric Respiratory and Sleep Medicine Women's and Children's Hospital Adelade South Australia Australia; ^5^ Department of Pediatrics MacKay Children's Hospital and MacKay Memorial Hospital Taipei Taiwan; ^6^ Department of Pediatrics Sibu Hospital Sibu Sarawak Malaysia; ^7^ Department of Pediatrics, Wan Fang Hospital Taipei Medical University Taipei Taiwan; ^8^ Department of Pediatrics Soroka University Medical Center Beer‐Sheva Israel; ^9^ Department of Pediatrics Selayang Hospital Batu Caves Selangor Malaysia; ^10^ Department of Pediatrics Chang Gung Children's Hospital, Linkou Chang Gung Memorial Hospital Taoyuan Taiwan; ^11^ Departments of Emergency Medicine and Children's Critical Care Gold Coast University Hospital Gold Coast Queensland Australia; ^12^ Department of Pediatric Infectious Diseases Çukurova University Faculty of Medicine Balcalı Turkey; ^13^ Department of Pediatric Pulmonology Ruth Rappaport Children's Hospital Haifa Israel; ^14^ Department of Pediatrics Hospital Tengku Ampuan Rahimah Klang Selangor Malaysia; ^15^ Department of Pediatrics University Malaya Medical Center Kuala Lumpur Malaysia; ^16^ Division of Pediatric Pulmonology Marmara University Istanbul Turkey; ^17^ Ark Biopharmaceutical Shanghai China; ^18^ Children's Foundation Research Institute Le Bonheur Children's Hospital Memphis Tennessee USA

**Keywords:** AK0529, fusion inhibitor, infants, respiratory syncytial virus (RSV), ziresovir

## Abstract

**Background:**

Respiratory syncytial virus (RSV) infection is a cause of substantial morbidity and mortality in young children. There is currently no effective therapy available.

**Methods:**

This was a Phase 2 study of the oral RSV fusion protein inhibitor AK0529 in infants aged 1–24 months, hospitalized with RSV infection. In Part 1, patients (*n* = 24) were randomized 2:1 to receive a single dose of AK0529 up to 4 mg/kg or placebo. In Part 2, patients (*n* = 48) were randomized 2:1 to receive AK0529 at 0.5, 1, or 2 mg/kg bid or placebo for 5 days. Sparse pharmacokinetic samples were assessed using population pharmacokinetics modelling. Safety, tolerability, viral load, and respiratory signs and symptoms were assessed daily during treatment.

**Results:**

No safety or tolerability signals were detected for AK0529: grade ≥3 treatment‐emergent adverse events occurring in 4.1% of patients in AK0529 and 4.2% in placebo groups, respectively, and none led to death or withdrawal from the study. In Part 2, targeted drug exposure was reached with 2 mg/kg bid. A numerically greater reduction in median viral load with 2 mg/kg bid AK0529 than with placebo at 96 h was observed. A −4.0 (95% CI: −4.51, −2.03) median reduction in Wang Respiratory Score from baseline to 96 h was observed in the 2 mg/kg group compared with −2.0 (95% CI: −3.42, −1.82) in the placebo group.

**Conclusions:**

AK0529 was well tolerated in hospitalized RSV‐infected infant patients. Treatment with AK0529 2 mg/kg bid was observed to reduce viral load and Wang Respiratory Score.

**Clinical Trials Registration:**

NCT02654171.

## 1 INTRODUCTION

Respiratory syncytial virus (RSV) is estimated to annually cause 30 million respiratory infections world‐wide, particularly in young children, with around three million associated hospital admissions and >60,000 in‐hospital deaths in children younger than 5 years.^1^ Safe and effective easily dosed therapies are largely absent. Monoclonal antibodies in development may provide certain protection from infection to infants,^2^ but for those infected, the standard of care is currently limited to supportive therapy.

A promising target for RSV drug development is the class I viral trimeric fusion (F) glycoprotein, which mediates RSV entry in response to binding of the attachment (G) glycoprotein to a host receptor. Several molecules targeting F protein have been evaluated in clinical challenge models in adults as well as in children with an acute RSV infection but with limited success.^3^, ^4^, ^5^, ^6^ Other compounds, such as RSV replication inhibitors, have also been tested in early stage clinical trials.^7^, ^8^

AK0529 (ziresovir) is a potent, selective, and orally bioavailable RSV F protein inhibitor.^9^ Cytopathic and plaque reduction assays have demonstrated antiviral activity with EC_90_ at nanomolar concentrations against all clinical RSV isolates (31 A‐subtypes and 29 B‐subtypes) collected on different continents during different infection seasons. In vivo, orally administered AK0529 has demonstrated antiviral efficacy in the BALB/c mouse RSV viral challenge model. Extensive preclinical studies on AK0529 suggested a suitable profile for development in human. Phase 1 trials in Australia and China and a human mass balance study in the UK showed good tolerability in multiple doses up to 300 mg bid in healthy adult volunteers (see Supporting Information).

Based on this profile, we performed a two‐part proof‐of‐concept Phase 2 study in infants aged 1–24 months hospitalized with RSV infection.

## 2 METHODS

### 2.1 Patient selection

Study participants were infants aged ≥1–≤24 months, weighing >3 kg at screening, and within the 10th and 90th age percentiles (inclusive), requiring hospitalization for management of virologically confirmed RSV infection. They were continuously enrolled from May 2016 to April 2019 RSV seasons in the subtropical regions of Australia, Taiwan, Malaysia, Turkey, and Israel.

### 2.2 Study design

This was a double‐blind, placebo‐controlled, randomized, multicenter study with the primary objective to evaluate the safety and tolerability of single and multiple oral doses of AK0529. Secondary objectives were to evaluate the effect of AK0529 on Wang Respiratory Score, to determine the effects of treatment on viral load, and to characterize the pharmacokinetics (PK) of single and multiple AK0529 doses.

The ethics committee at each trial center approved the trial. All subjects' parents or legal guardians provided written informed consent.

The study consisted of two parts. In both parts, subjects were randomized in a 2:1 ratio to receive active treatment or placebo. Subjects were stratified by age (6 to 24 months in Cohorts 1 and 3, and 1 to <6 months in Cohorts 2 and 4).

Part 1 was a single‐dose study with a 7‐day follow‐up. The doses administered to patients in Cohort 1 included 4, 2, and 1 mg/kg. The Safety Review Committee (SRC) reviewed PK and safety data after each of the sets of three patients, before further enrollment of subjects, and recommended 0.5 and 1 mg/kg as the doses for patients in Cohort 2.

In Part 2, subjects received AK0529 or placebo over 5 days and were followed until day 14 post‐initial dose. The doses in Cohort 3 included 1 and 0.5 mg/kg bid. In Cohort 4, the doses included 2, 1 and 0.5 mg/kg bid.

Details of the rationale for the dose selection, subject dosing, and safety review schemes in Parts 1 and 2 are detailed in the Supporting Information.

### 2.3 Study assessments

The Wang Respiratory Score assesses the severity of respiratory rate, wheezing, retraction of respiratory muscles, and general condition on a scale from 0 to 3 except for the general condition, which is scored 0 for normal and 3 for irritability or lethargy. The sum score, ranging between 0 and 12 (most severe), reflects the severity of the respiratory system disorders in children.^10^ Viral load and viral load area under the curve (AUC) were determined on viral specimens collected through either nasopharyngeal swab or aspirate methods by quantitative reverse‐transcription PCR in duplicate.^11^ Amino acid substitutions related to potential development of drug resistance^12^ were monitored by Sanger, sequencing the RSV *F* gene from clinical specimens collected at the baseline and the last visit.

### 2.4 Safety

Safety and tolerability was evaluated by clinical assessment of safety data, including laboratory data, electrocardiographic data, vital signs, and adverse events (AEs).

### 2.5 PK

Individual plasma concentrations of the active drug were measured, and PK were determined using nonlinear mixed‐effect models. In all cases, when new data were available, the updated model was used for evaluation under the assumption of an allometric coefficient of 0.75 and incorporation of CYP maturation. Using a Population PK (Pop‐PK) analysis approach, AUC_0–∞_, clearance (CL/F), volume of distribution (Vc/F), and other PK parameters were estimated for AK0529. Maximum plasma concentration (C_max_) was computed from the predicted profiles.

### 2.6 Statistical analysis

Clinical safety and tolerability were assessed on the safety population, comprising all subjects who received ≥1 dose of study drug. Clinical variables were evaluated in the full analysis population, which included all subjects who received a dose of study drug and for whom ≥1 post‐treatment assessment was available. Pharmacokinetic analysis was performed on those subjects in the safety population for whom both pre‐ and post‐dose samples were available.

All data are summarized by descriptive statistics with 95% confidence intervals for comparisons. As respiratory viral infections may coexist with potentially pathogenic respiratory bacteria that may affect the severity of RSV infection,^13^, ^14^ a post‐hoc analysis of clinical efficacy was performed, excluding patients with bacterial pneumonia diagnosed by the treating physicians. There was no general screening for bacterial co‐infection.

In study Part 1, no formal sample size calculation was performed. For Part 2, it was calculated that a sample size of 16 patients in each active treatment group would provide >90% power to detect a 50% reduction (mean ratio of 0.5) in RSV viral load AUC with AK0529 as compared with placebo, using a one‐sided significance level of 0.05, two‐sample equal variance *t*‐test, and assuming a 0.3 variation coefficient of the placebo for viral load. These assumptions were based on viral challenge models of early treatment in adults.^3^ For the transaminase shift analysis, the Chi‐square test and Fisher's exact test were used, as appropriate, to test for differences between active treatment and placebo.

All analyses were performed using SAS® Version 9.3 (SAS Institute Inc., Cary, NC, USA).

## 3 RESULTS

### 3.1 Study population

**FIGURE 1:**
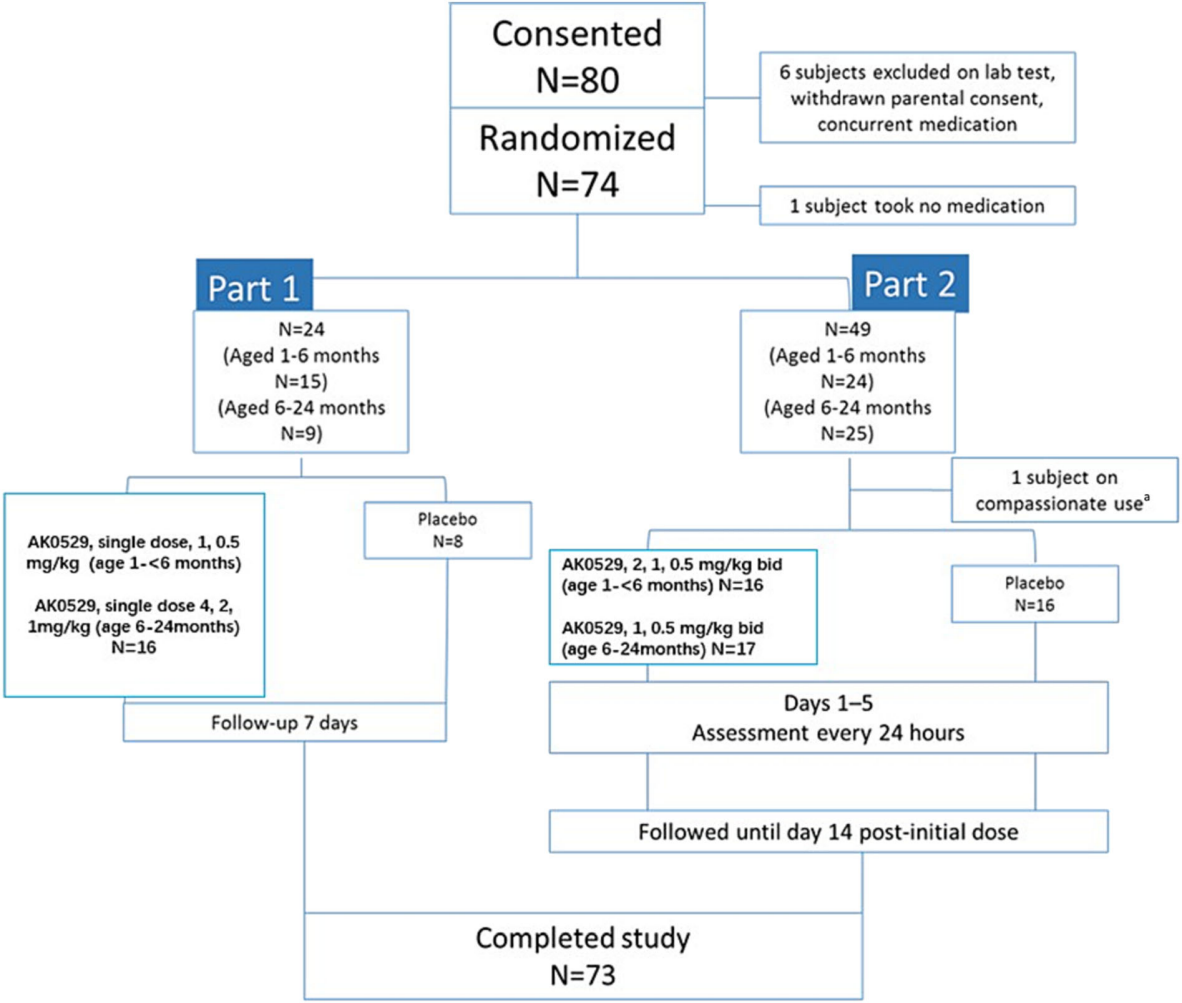
Study flow chart. ^a^One randomized patient in Part 2 received unblinded treatment with AK0529 (2 mg/kg bid) on a compassionate use basis for the best interest of the patient and was excluded from the efficacy analysis unless specified.

The study flow is shown in Figure 1. Seventy‐three subjects were enrolled in the study, including 20 subjects from Australia, 39 from Taiwan, eight from Malaysia, three from Israel, and three from Turkey. Detailed distribution of subjects in each country or region by age group and gender is provided in the Supportive Information. One randomized subject in Part 2 did not receive study medication because of early discharge by the study site and was excluded from all analyses. One subject in Part 2 received unblinded treatment with AK0529 (2 mg/kg bid) on a compassionate basis because of the late arrival of the study medication at the study center. The subject was excluded from the efficacy analysis unless otherwise specified. Accordingly, the full analysis population consisted of 72 subjects (Figure 1). Baseline characteristics and viral load and Wang Respiratory Score are shown in Tables 1 and 2, respectively. Virological samples were available for analysis in 71 subjects. In Part 1, 10 subjects were infected with RSV‐A and 13 with RSV‐B; whereas in Part 2, 23 subjects were infected with RSV‐A and 25 with RSV‐B.

**TABLE 1:** Demographic characteristics. Percentages do not always add up to 100% because of rounding.

Characteristic	AK0529 (*N* = 48)	Placebo (*N* = 24)	Total (*N* = 72)^a^
Age: Overall (months) mean ± SD	7.32 ± 4.72	8.10 ± 6.33	7.58 ± 5.27
Age 6–24 (months) mean ± SD	10.92 ± 3.07	12.40 ± 5.63	11.40 ± 4.06
Age: 1–< 6 (months) mean ±SD	2.90 ± 1.44	3.02 ± 1.25	2.94 ± 1.36
Male sex, n (%)	34 (71%)	11 (46%)	45 (62%)
Ethnicity, n (%)			
Caucasian	14 (29%)	9 (37.5%)	23 (32%)
Asian	32 (67%)	15 (62.5%)	47 (65%)
Aboriginal	1 (2%)	0	1 (1%)
Other	1 (2%)	0	1 (1%)
Weight (kg) mean ± SD	7.71 ± 2.10	7.34 ± 2.45	7.59 ± 2.21
Length (cm) mean ± SD	66.18 ± 6.66	66.95 ± 9.38	66.43 ± 8.85
Head circumference (cm) mean ± SD	42.78 ± 3.22	42.61 ± 3.69	42.72 ± 3.36

^a^ One subject received unblinded treatment with AK0529 (2 mg/kg bid) on a compassionate use basis and was excluded from the full analysis population.

**TABLE 2:** Baseline Wang respiratory score and viral load in the groups treated with different doses of AK0529 or placebo.

	AK0529 0.5 mg/kg	AK0529 1.0 mg/kg (per day for Part 2)	AK0529 2.0 mg/kg (per day for Part 2)	AK0529 4.0 mg/kg (per day for Part 2)	Placebo
Baseline Wang Respiratory Score, median (min, max)					
Part 1	6.0 (2, 8) (*n* = 4)	4.5 (3, 7) (*n* = 6)	5.5 (3, 8) (*n* = 4)	9.0 (8, 10) (*n* = 2)	5.0 (2, 9) (*n* = 8)
Part 2	‐	5.5 (2, 8) (*n* = 10)	5.0 (1, 11) (*n* = 11)	5.0 (2, 9) (*n* = 11)	5.0 (2, 8) (*n* = 16)
Baseline viral load (Log_10_ PFUe/mL) median (min, max)					
Part 1	5.75 (3.06, 6.58) (*n* = 4)	4.99 (4.66, 5.77) (*n* = 6)	5.00 (3.49, 7.11) (*n* = 4)	4.16 (3.46, 4.86) (*n* = 2)	4.62 (0.47, 7.09) (*n* = 8)
Part 2	‐	4.22 (2.61, 6.32) (*n* = 10)	5.02 (3.40, 7.19) (*n* = 11)	5.09 (2.98, 7.28) (*n* = 11)	4.70 (3.46, 8.11) (*n* = 16)

### 3.2 Reduction in viral load and S&S scores

**FIGURE 2:**
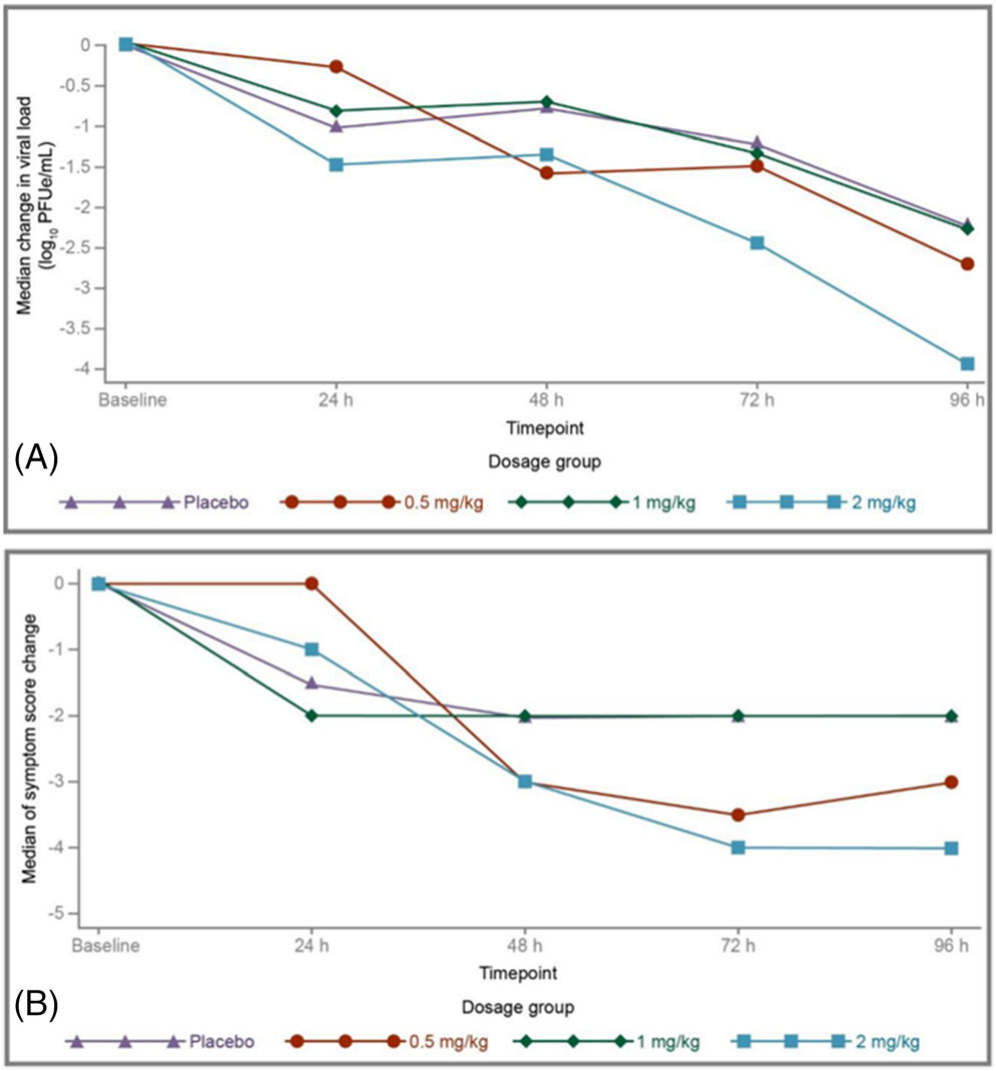
(A) Median change in viral load from baseline to 96 h post‐dose in Part 2 (full analysis population). (B) Median change in Wang respiratory score by dose level in Part 2 (full analysis population). *p < 0.05 for the difference between the 2 mg/kg and placebo groups on Wang respiratory score reduction at 96 h.

In Part 1, reductions in viral load at 24 h after a single dose AK0529 administration were <1 log_10_ PFUe/mL relative to placebo. In Part 2, the multiple 2 mg/kg bid dose was associated with 1.25 log_10_ PFUe/mL greater reductions in median viral load than placebo at 72 h and 1.73 log_10_ PFUe/mL greater reductions at 96 h post‐dose (Figure 2A). These differences are not statistically significant.

**FIGURE 3:**
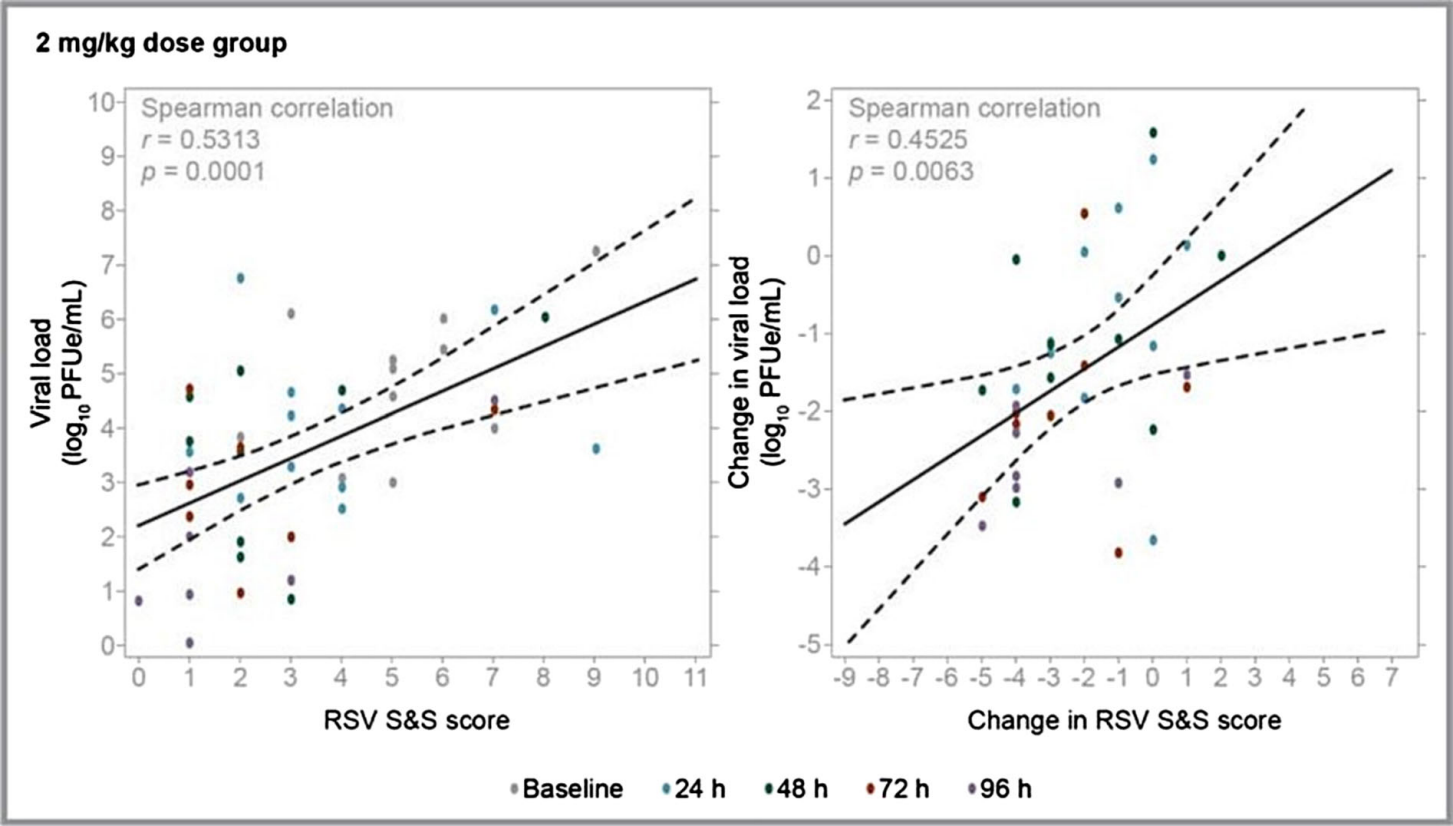
(A) Relationships between viral load and Wang respiratory score: viral load vs. RSV S&S score, *p* = 0.0001; interception: 95% CI: 1.34–2.92; slope: 95% CI: 0.23–0.61. (B) Relationships between changes in viral load and in Wang respiratory score over time: change in viral load vs. change RSV S&S score, *p* = 0.006; interception: 95% CI: −1.52– −0.22; slope: 95% CI: 0.06–0.51.

In Part 1, median Wang Respiratory Score decreased from baseline to 24 h in a dose‐related manner (see plot in the Supporting Information), with no change in the placebo group (one‐sided *p* = 0.004 for the combined AK0529 groups vs. placebo using the Wilcoxon rank–sum test). In Part 2, there was a −4.0 median reduction (95% CI: −4.51, −2.03) from baseline to 96 h in Wang Respiratory Score in the 2 mg/kg AK0529 group compared with −2.0 (95% CI: −3.42, −1.82) in the placebo group (median two‐sample test one‐sided exact p = 0.031, Hodges–Lehmann estimation −1 [95% CI, −2, 1] for the between‐group difference; Figure 2B). The differences in Wang Respiratory Score were greater when two cases of bacterial pneumonia (one in the 2 mg/kg AK0529 group and one in the placebo group, both of which were confirmed microbiologically or clinically) were excluded (Hodges–Lehmann estimation −1 [95% CI, −2, 0]; median one‐sided exact two‐sample test *p* = 0.009). In the full analysis population of both Parts 1 and 2 of the study, statistically significant correlations between viral load and Wang Respiratory Score included raw values and changes from baseline within the 2 mg/kg bid AK0529 group (Figure 3).

In a post‐hoc analysis, 8/11 patients (73%) receiving AK0529 2 mg/kg bid achieved disease remission by Day 5, as defined by Wang Respiratory Score ≤1. In contrast, only 5 out of 16 (31%) in the placebo group (one‐sided *p*‐value for the comparison 0.0412; Fisher's exact test) achieved disease remission.

Sanger sequencing on *F* gene was performed for all 133 RSV positive viral specimen collected at the baseline and the last visits of subjects. Among them, one specimen collected at 96 h post‐dose in one subject (2.7%) who was treated with 2 mg/kg bid AK0529 detected a mixed T400I mutation in the RSV‐A *F* gene. No associated clinical virological failure was detected. T400 is located in the intervening region between HR1 and HR2 of *F* protein, and its T400I mutation was known to cause a resistance to an RSV fusion inhibitor.^12^ We prepared recombinant RSV T400I variant and confirmed its resistance to AK0529.

Coinfection with ≥1 other respiratory virus was identified in 9/22 analyzed patients in Part 1 (40.9%), including three (13.6%) with ≥2 additional respiratory viruses. In Part 2, ≥1 other respiratory viruses were identified in 13 of 41 analyzed patients, including ≥2 additional respiratory viruses in three patients (7.3%). Human rhinovirus was the most frequently co‐detected virus in both populations, followed by adenovirus C and adenovirus B/E.

### 3.3 Safety

The safety profile of AK0529 is shown in Table 3 (see Supporting Information for a detailed summary of treatment emergent AEs [TEAEs]). Three subjects (6%) in the AK0529 group experienced a Grade 1 TEAE, which is considered drug‐related by the investigator: one case each of increased aspartate aminotransferase (1 mg/kg bid, Cohort 3), increased transaminases (2 mg/kg bid, Cohort 3), and hyperkalemia (2 mg/kg bid Cohort 4). Analyzing all alanine transaminase (ALT) and aspartate transaminase (AST) shifts from baseline, derived from the laboratory listings, no association was found between exposure to active drug and any deterioration in transaminase levels. No statistically significant differences were observed between active and placebo groups in ALT and AST (*p* = 0.516 and *p* = 0.644, respectively; Fisher's exact test). The shifts evaluation for AST and ALT is shown in Table 4.

**TABLE 3:** Adverse event profile of AK0529 compared with placebo.

	AK0529 (*N* = 49), n (%)	Placebo (*N* = 24), n (%)	Total (*N* = 73), n (%)
Subjects with any AEs	25 (51.0%)	10 (41.7%)	35 (47.9%)
Subjects with any TEAEs	24 (49.0%)	10 (41.7%)	34 (46.6%)
Subjects with any drug related TEAEs	3 (6.1%)^a^	0	3 (4.1%)
Subjects with any serious TEAEs	2 (4.1%)^b^	0	2 (2.7%)
Subjects with any TEAEs induced withdrawal	0	0	0
Subjects with any grade ≥3 TEAEs	2 (4.1%)^c^	1 (4.2%)^d^	3 (4.1%)
Subjects with any TEAEs leading to death	0	0	0

Abbreviations: AEs, adverse events; SAEs, serious adverse events; TEAEs, treatment‐emergent adverse events.

^a^ One case each of increased aspartate aminotransferase, increased transaminases, hyperkalemia.

^b^ Two cases of unrelated pneumonia.

^c^ One case of pneumonia, classified as SAE; one case of elevated transaminase.

^d^ One case of pneumonia.

**TABLE 4:** Shifts evaluation for aspartate aminotransferase and alanine aminotransferase (safety population).^a^

Parameter	AK0529 (N = 39) n (%)	Placebo (N = 16) n (%)	*p*‐value
AST (aspartate aminotransferase)			
n	39	14	0.2939
Normal to abnormal CS	2 (5.1%)	0	‐
Normal to abnormal NCS	4 (10.3%)	0	‐
Abnormal NCS to abnormal CS	0	0	‐
No change	29 (74.4%)	12 (85.7%)	‐
Abnormal NCS to normal	2 (5.1%)	2 (14.3%)	‐
Abnormal CS to abnormal NCS	1 (2.6%)	0	‐
Abnormal CS to normal	1 (2.6%)	0	‐
ALT (alanine aminotransferase)			
n	38	15	0.5422
Normal to abnormal CS	1 (2.6%)	1 (6.7%)	‐
Normal to abnormal NCS	1 (2.6%)	1 (6.7%)	‐
Abnormal NCS to abnormal CS	1 (2.6%)	0	‐
No change	34 (89.5%)	12 (80.0%)	‐
Abnormal NCS to normal	1 (2.6%)	1 (6.7%)	‐
Abnormal CS to abnormal NCS	0	0	‐
Abnormal CS to normal	0	0	‐

Abbreviations: CS, clinically significant; *N*, number of subjects in the population; *n*, number of subjects in the specified category; NA, not applicable; NCS, not clinically significant.

^a^ For a subject with multiple shifts with different categories, only the worst shift category was counted, where the hierarchy for shift category was ordered from “Normal to abnormal CS” (worst) to “Abnormal CS to normal” (best). Cochran–Mantel–Haenszel test was used to calculate the *p*‐value.

Two serious TEAEs (pneumonia) were reported: one of Grade 4^15^ (also counted as a serious AEs [SAE], 2 mg/kg bid, Cohort 4) and one Grade 2 (0.5 mg/kg bid, Cohort 4). The relationships to study treatment were respectively considered “not related” and “unlikely”, and both AEs were resolved without sequelae.

### 3.4 PK

Pharmacokinetic parameters evaluated in Part 1 are shown in Table 5. There was a dose‐dependent increase in AUC with no apparent plateau up to 4 mg/kg. Bioavailability was high (up to AUC_0–24_, up to 7780 h.ng/mL). C_max_ ranged from 158 ng/mL with 0.5 mg/kg to 375 ng/mL with 2.0 mg/kg dose, comfortably above the targeted 5 × EC_90_ value of 40 ng/mL. With the 4.0 mg/kg dose, C_max_ reached 888 ng/mL, which exceeded the predefined safety limit. The time to reach C_max_ was around 4 h, except for the 4 mg/kg dose that peaked at around 2 h. The elimination half‐life was slightly longer in subjects aged <6 months.

**TABLE 5:** Predicted concentration‐time profiles for different single doses of AK0529 from Part 1 study.

Dose (mg/kg)	N	C_max_mean (CV %, ng/mL)	t_max_median (range, h)	AUC_0–∞_mean (CV %, h.ng/mL)	AUC_0–24_ mean (CV %, h.ng/mL)	t_½_mean (CV %, h)
0.5	4	158 (72.2)	4.5 (3.5, 7.0)	2060 (49.6)	1770 (56.9)	7.7 (27.7)
1.0	6	302 (97.6)	4.3 (1.8, 7.5)	3150 (101)	2890 (100)	5.7 (33.5)
2.0	4	375 (50.6)	3.3 (2.0, 7.0)	4200 (49.5)	3790 (47.4)	6.2 (20.2)
4.0	3^a^	888 (58.6)	2.5 (2.0, 2.5)	8280 (47.2)	7780 (50.5)	6.0 (26.4)

*Note*: All subjects receiving 0.5 mg/kg dose were younger than 6 months.

Abbreviations: AUC, area under the plasma concentration‐time curve; Cmax, maximal concentration; CV, coefficient of variation; t_1/2_, elimination half‐life; t_max_, time to reach maximum.

^a^ The 4 mg/kg dose group includes additional data from a single subject treated in a preceding phase 1b study (Study AK0529‐1002).

In Part 2, an approximate dose proportionality was observed for mean C_max_ and AUCτ across the dose range tested. A slight increase (approximately 1.5‐fold) in AUCτ from Days 1 to 5 indicates low‐to‐moderate drug accumulation following bid dosing. The median t_max_ was comparable between Days 1 and 5 (2.5–3.8 h), with a median trough of 12 h.

## 4 DISCUSSION

The current Phase 2 study supports a favorable safety profile of the *F* protein inhibitor AK0529 in infants aged 1–24 months hospitalized with RSV infection. The rate of grade ≥3 TEAEs was 4.1% compared with 4.2% in the placebo groups, and none led to study withdrawal. In addition, there was a potentially positive effect of AK0529 on Wang Respiratory Score, as well as a numerically greater effect on RSV viral load. The pharmacokinetic analysis indicated a rapid, dose‐dependent achievement of efficacious plasma drug concentrations and good bioavailability. Clinical improvement rates with the 2 mg/kg bid dose were substantially higher than with placebo.

The results may appear moderate but need to be viewed in context. In a Phase II study with JNJ‐8678 in infants hospitalized with RSV, a greater reduction in RSV viral load than with placebo did not translate into observed clinical benefit, with no improvements in length of hospital stay or time to clinical stability.^16^ Similar difficulties have been encountered in studies of COVID‐19 treatments. In the BLAZE‐1 trial, there appeared to be no correlation between effects on viral load from different doses of the neutralizing antibody LY‐CoV555 and reductions in the need for hospitalization.^17^ Remdesivir, although a potent inhibitor of SARS‐CoV‐2 virus replication in cell cultures, has been reported to have no effect on viral load in patients with severe COVID‐19, although a numerically shorter time to clinical improvement was observed.^18^ Against such a background, the effects on RSV viral load and signs and symptoms with AK0529 appear promising and support further clinical investigation. We included the proportion of patients who achieved Wang Respiratory Score ≤1, or disease remission, at the end of treatment in a post‐hoc assessment, an endpoint that has been used in antiviral drug trials, as well as in the evaluation of novel therapies against SARS‐CoV‐2.^19^

The safety profile of AK0529 was favorable, an important consideration given the age of the studied population. The two cases of pneumonia were deemed unrelated to the treatment. Increased transaminase level is associated with the disease severity of RSV infection,^20^, ^21^, ^22^ but we did not find association between active drug exposure and any deterioration in transaminase levels. The elevations observed most likely reflected the disease severity of RSV bronchiolitis in the infants.

A problem with some candidate molecules has been the resistance mutations in RSV strains.^4^ Only one indication of resistance to AK0529 was detected in a single subject. It is debatable whether resistance is a serious concern in immune‐competent subjects with a pathogen such as RSV, which has high replication rates but is rapidly cleared by the body.^23^ Vigilance is nevertheless warranted.

Although of limited size, this Phase 2 study enrolled patients from five countries/regions on three continents during RSV seasons in four calendar years, suggesting that the results are representative of global patients and treatment conditions. A disadvantage is that the time from infection to first dose could not be captured. It is difficult to determine the time of infection in infants who are not able to communicate. Moreover, some of the participating centers were central hospitals treating severely ill patients transferred from local clinics with possibly limited initial records. On the upside, the results support the efficacy of AK0529 under conditions closely related to those of actual clinical practice and regardless of time from infection to treatment.

Among other shortcomings, the limited follow‐up time meant that the question of rebound could not be addressed. The 96 h timepoint was not the pre‐defined primary timepoint for the analysis, and significance tests were not adjusted for multiple analyses. Differences in national healthcare and insurance policy prevented analysis of the potential impact of the therapy on the length of hospital stay. There was a limited information about the need for respiratory support (e.g., ventilation or nasal oxygen) at baseline, which may have affected the assessment of disease severity. There is an ongoing discussion around the relative performance of different sampling methods for respiratory viruses.^24^

In conclusion, this proof‐of‐concept study supported a relevant effect on viral load and clinical signs and symptoms with 2 mg/kg bid AK0529 in infants hospitalized with RSV infection. Further study on AK0529 is warranted.

## AUTHOR CONTRIBUTIONS

**Li‐Min Huang**: Investigation; methodology; resources; supervision; validation; review and editing. **Andreas Schibler**: Investigation; methodology; resources; supervision; validation; review and editing. **Yi‐Chuan Huang**: Investigation; methodology; resources; supervision; validation. **Andrew Tai**: Investigation; methodology; resources; supervision; validation. **Hsin Chi**: Investigation; methodology; resources; supervision. **Chae‐Hee Chieng**: Investigation; resources; supervision. **Jinn‐Li Wang**: Investigation; resources; supervision. **Aviv Goldbart**: Investigation; resources; supervision. **Swee‐Ping Tang**: Investigation; resources; supervision. **Yhu‐Chering Huang**: Investigation; resources; supervision. **Shane George**: Investigation; resources; supervision. **Derya Alabaz**: Investigation; resources; supervision. **Lea Bentur**: Investigation; resources; supervision. **Siew‐Choo Su**: Investigation; resources; supervision. **Jessie de Bruyne**: Investigation; r, supervision. **Bulent Karadag**: Investigation; resources; supervision. **Feng Gu**: Data curation; formal analysis; project administration; software; validation; visualization; writing. **Gang Zou**: Data curation; formal analysis; project administration; validation; visualization. **Stephen Toovey**: Conceptualization; data curation; formal analysis; methodology; resources; supervision; validation; review and editing. **John P. DeVincenzo**: Data curation; formal analysis; investigation; methodology; validation; review and editing. **Jim Z. Wu**: Conceptualization; data curation; formal analysis; funding acquisition; methodology; resources; supervision; validation; writing.

## CONFLICT OF INTEREST STATEMENT

JZW is a co‐inventor of patents (WO2013020993A, 2012; CN105726488B, 2014) covering a compound targeting RSV diseases and a preparation method of the formula. ST and JZW are co‐inventors of a patent (WO2021083290A1; 2020) covering RSV fusion protein inhibitor composition and its use for the treatment and prophylaxis of RSV. FG, ST, GZ, and JZW are or were employees of and are shareholders in Ark Biosciences. JPD served as a compensated scientific consultant for Ark Biosciences and is a shareholder in the company. All other authors declare no competing interests.

## ETHICS APPROVAL STATEMENT

The ethics committee at each trial center approved the trial. All subjects' parents or legal guardians provided written informed consent. All subjects' parents or legal guardians provided written informed consent.

## Supporting information


**Data S1.** Supporting Information.

## Data Availability

As this is a study in pediatric patients, the raw data are not publicly available because of privacy and ethical restrictions.
