# Supplementary material for: Safety and efficacy of AK0529 in respiratory syncytial virus‐infected infant patients: A phase 2 proof‐of‐concept trial
Source: Influenza Other Respir Viruses. 2023 Jul 25;17(7):e13176. doi: 10.1111/irv.13176 (PMC10368966; doi:10.1111/irv.13176)
Supplement: Supplementary file 1 — Data S1. Supporting Information. [file IRV-17-e13176-s001.docx]

# Supporting Information

1. Slide deck of AK0529’s Phase 1 Study Results Presented at 1st International Meeting on Respiratory Pathogens in Singapore in 2015

p2-9

1. Details of Phase 2 study design including the rationale for dose selection,

subject dosing/safety review schemes, subject distribution in different countries, age and gender, and enrollment inclusion and exclusion criteria

p10-14

1. Change of Wang Respiratory Score at 24 hours after a single dose of AK0529 in Phase 2 Part 1 Study

p15

1. Summary of TEAEs by preferred MedDRA terms and system organ class in the Phase 2 study

p16-17

1. Analysis of ALT and AST changes during treatment

p18

1. **AK0529’s Phase 1 Study Results Presented International Meeting on Respiratory Pathogens in Singapore in 2015**


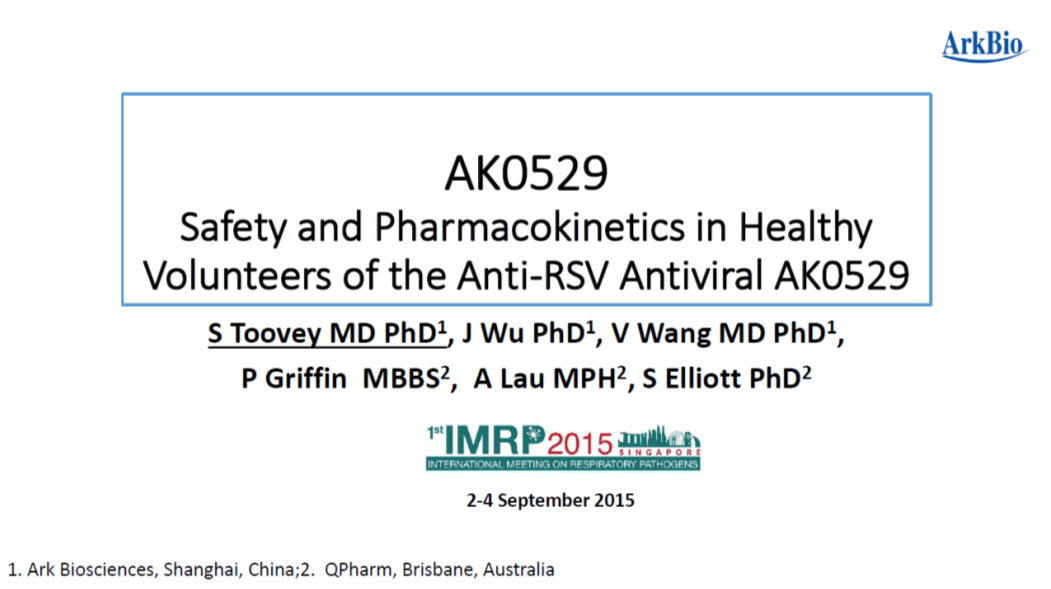


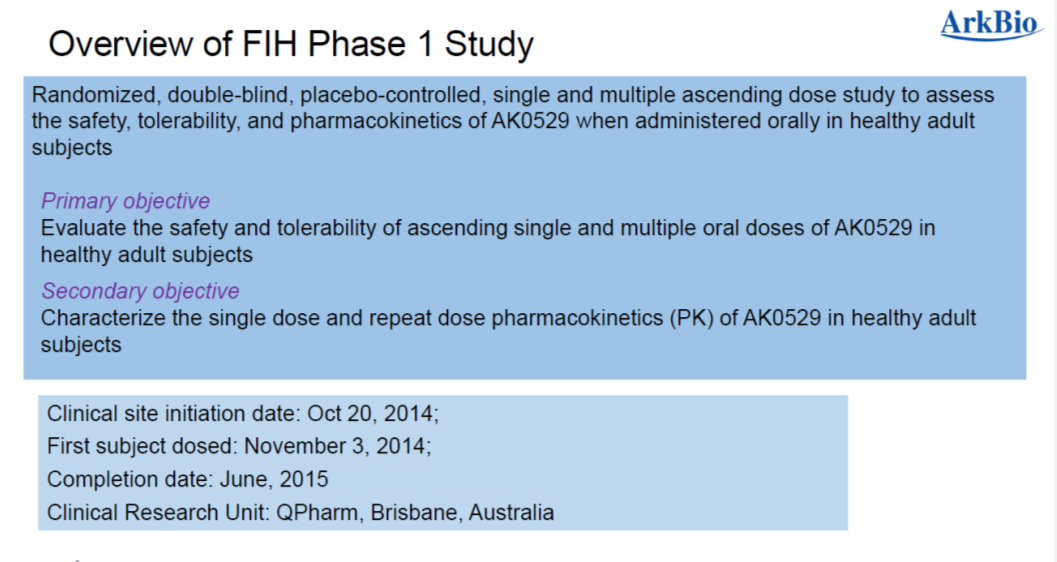


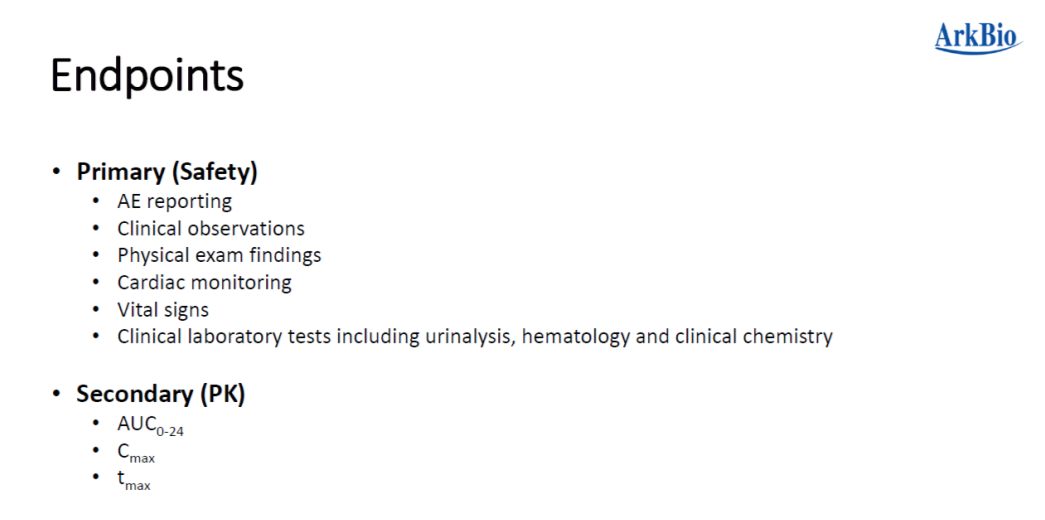


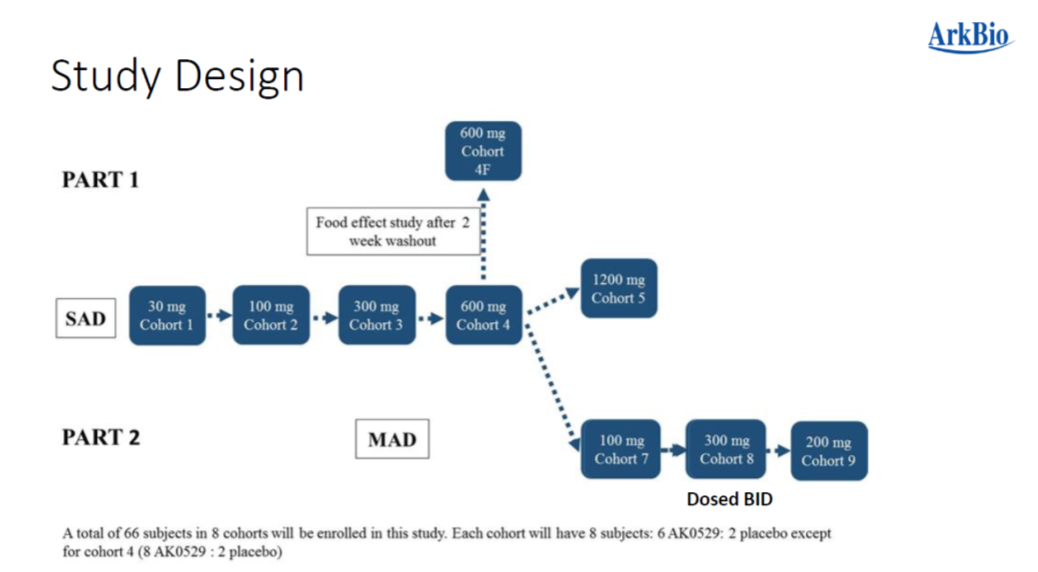


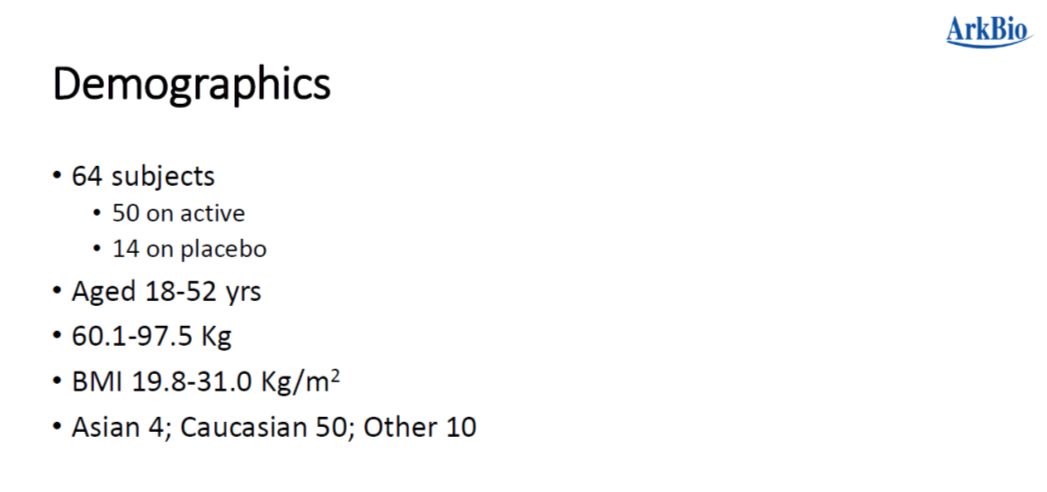


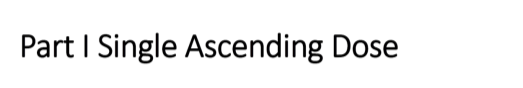


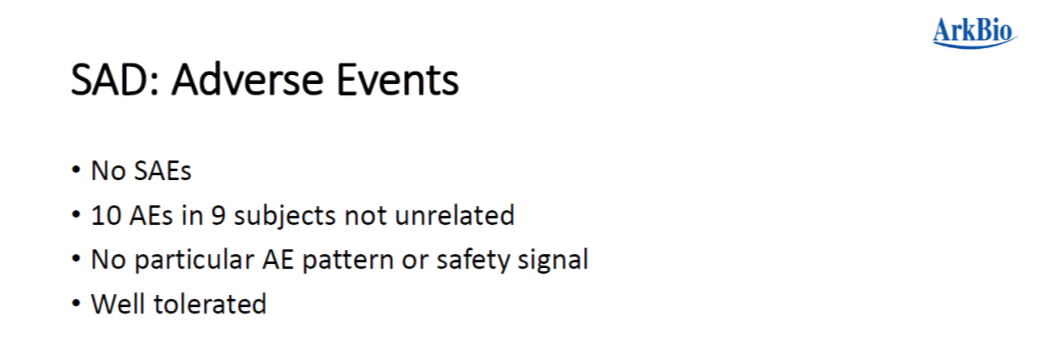


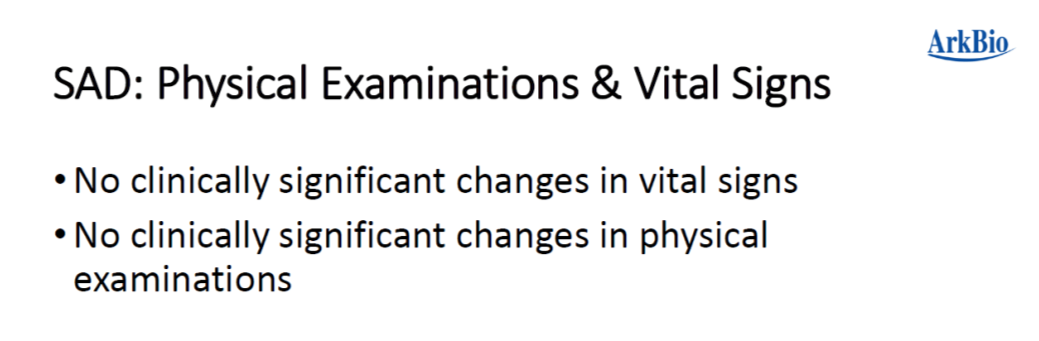


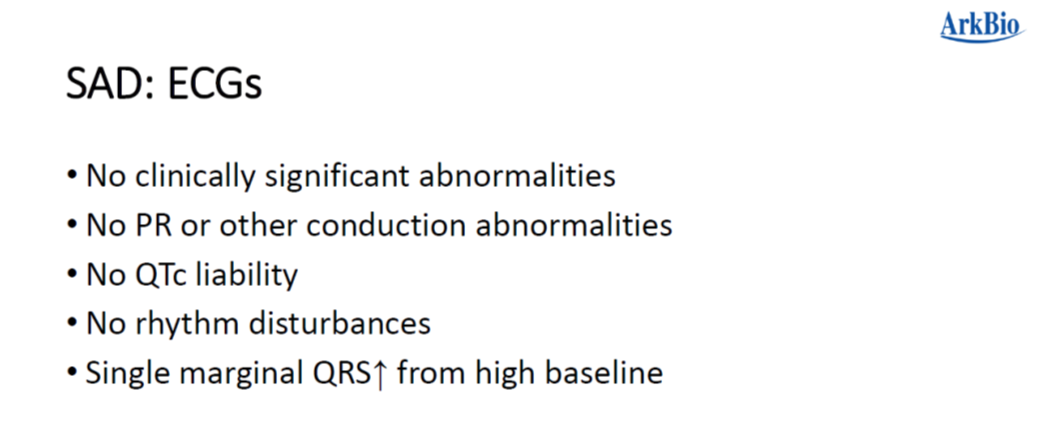


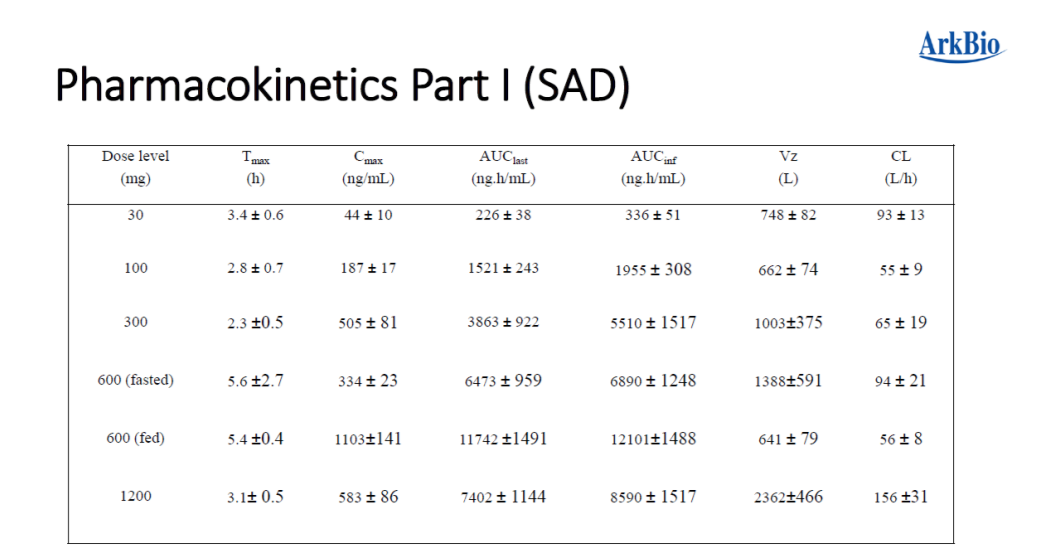


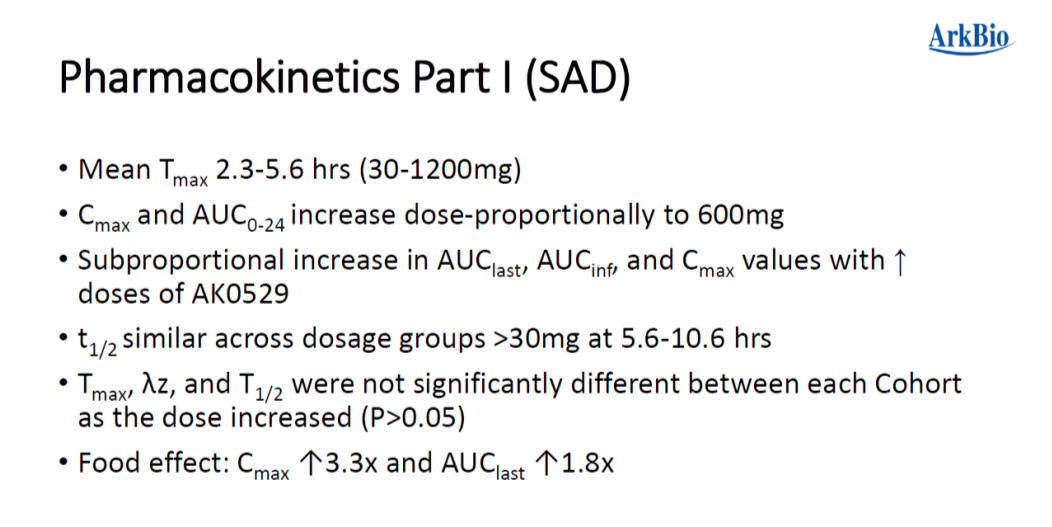


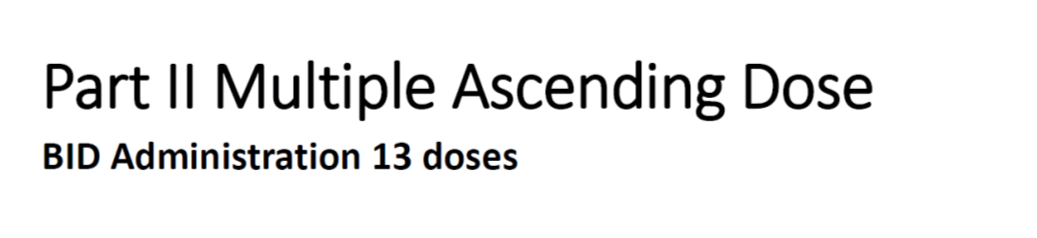


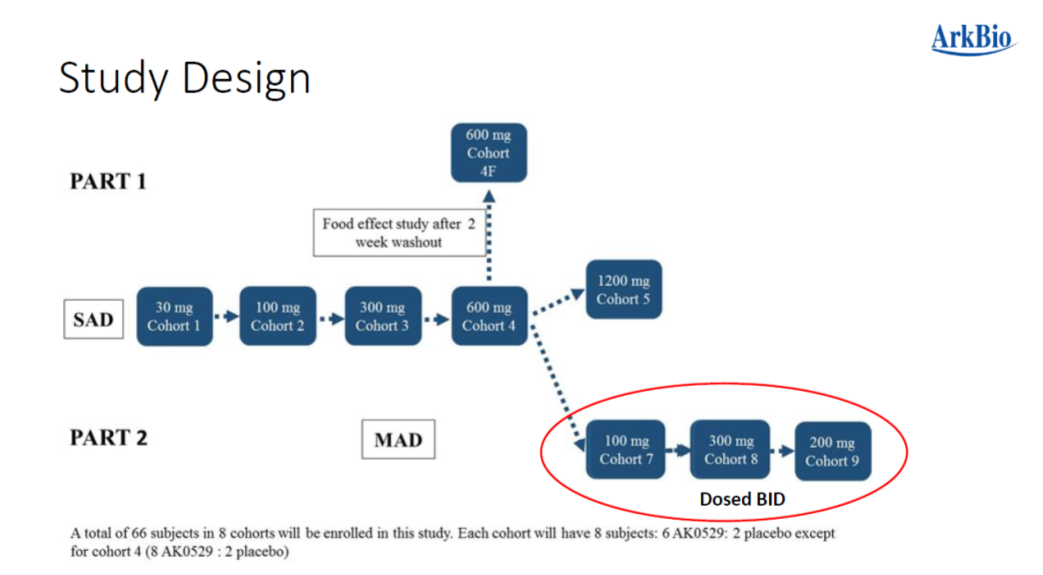


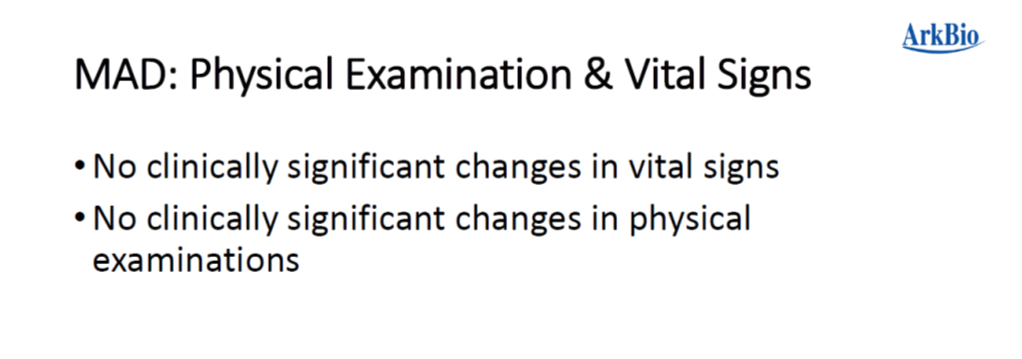


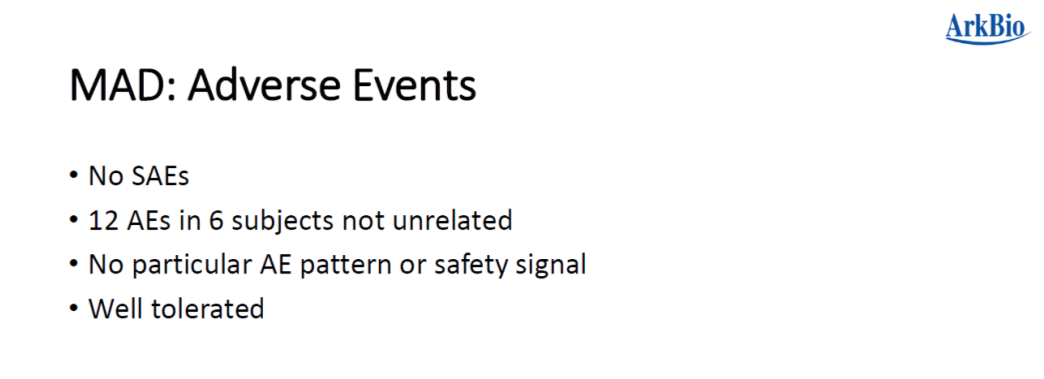


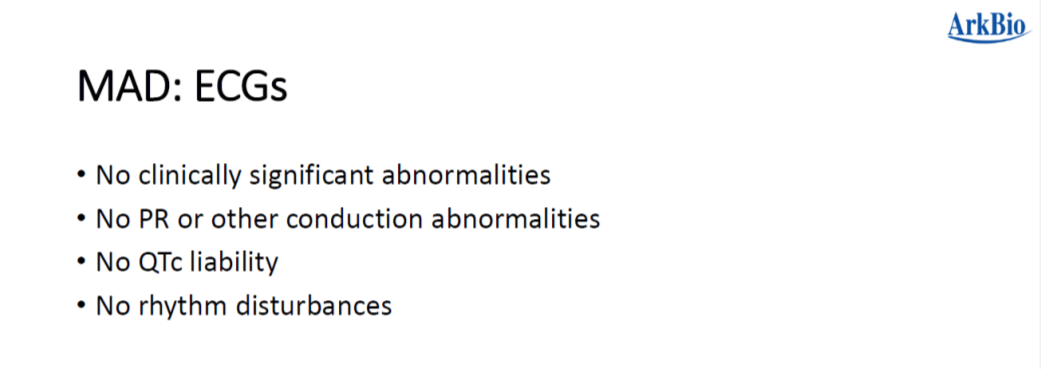


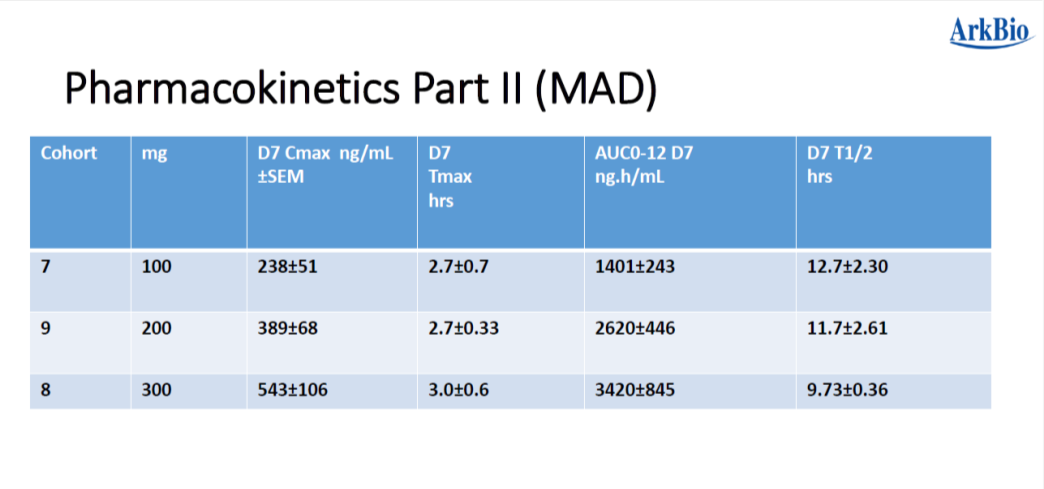


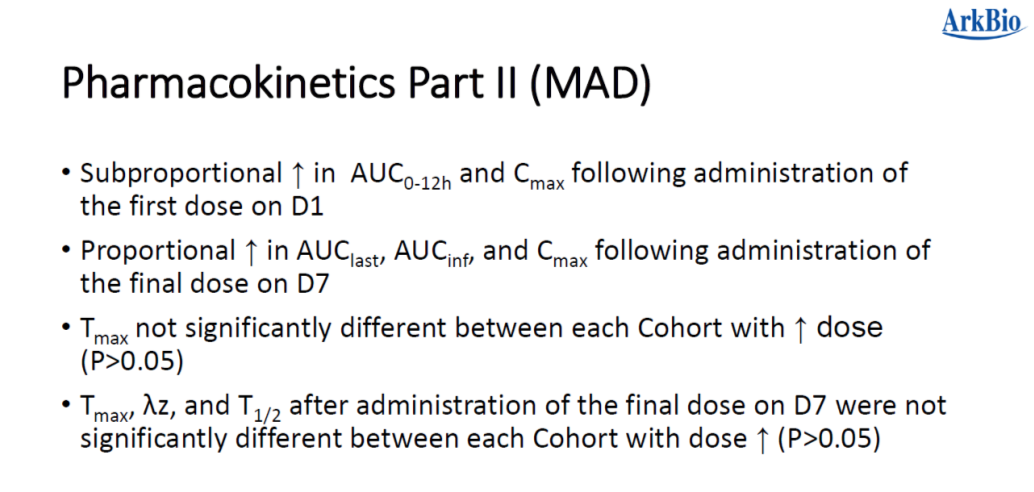


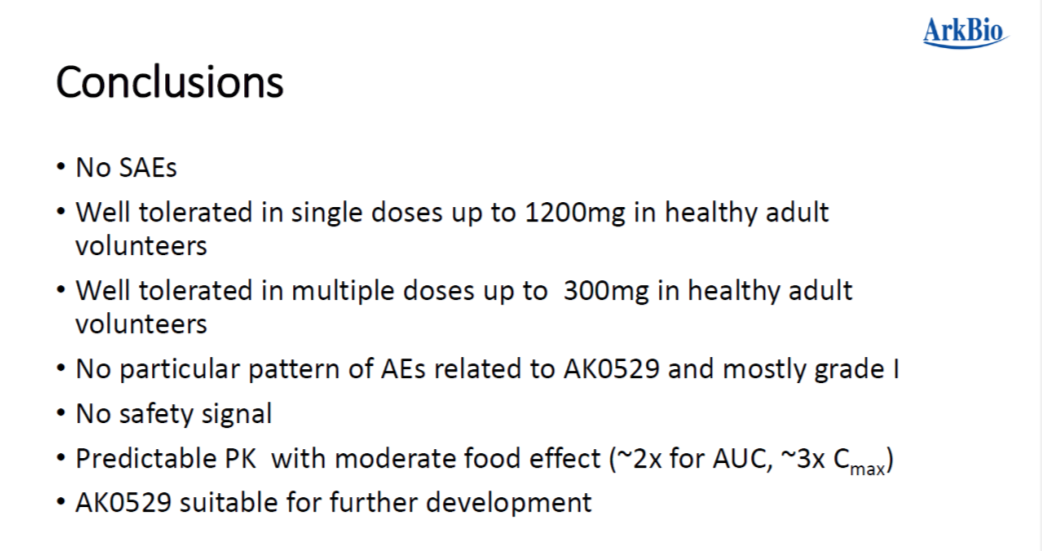


1. **Details of Phase 2 study design including the rationale for dose selection, subject dosing/safety review schemes, and patient inclusion and exclusion criteria**

## Phase 2 Study Design

The Safety Review Committee (SRC) included the primary investigator, an independent paediatrician, a pharmacokineticist, and a physician representative of the sponsor. Pharmacokinetic and pharmacodynamic modelling for dose optimisation was performed by Model Answers (Brisbane Australia) on unblinded clinical data. In part 1, The SRC reviewed the full safety and pharmacokinetic data after the first three infant patients in Cohort 3 had completed dosing of a minimum of three infant patients, before enrollment continued in Cohort 3 and was initiated in Cohort 4. The same SRC procedure was followed for the first three infant enrolled in Cohort 4 and the next six infant patients enrolled in Cohort 3. After SRC approval of the data for the first nine infant patients in each cohort, the final 15 infant patients per cohort were recruited.

## Rationale for Dose Selection

Non-clinical data from cytopathic effect (CPE) assays using Hep-2 cells showed that AK0529 has potent dose-dependent antiviral activity against laboratory strains of both human RSV subtype viruses, RSV-A and RSV-B. The average concentration of at which a 90% inhibition (EC_90_) of the RSV-induced reduction of cell viability was observed was 5 nM (2.2 ng/mL). The potency remained the same when AK0529 was tested against various RSV clinical isolates. The EC_90_ value was determined in the presence of the known plasma protein binding in the CPE assay. Adjusting for the free drug fraction ratio in human and mouse plasma and molecular weight (439.5 g/mol), the EC_90_ value was determined as 7.9 ng/mL in human plasma. To achieve a target efficacious concentration in humans, it was expected that a minimum target of 5 × EC_90_ value, or approximately 40 ng/mL should be attained. To maintain the effectiveness, this target C_trough_ level was required to be achieved across the dosing interval. In healthy adult subjects, single AK0529 doses of 100 mg and above achieved mean plasma concentration levels >50 ng/mL at 12 hours post-dose, suggesting the efficacious dose of AK0529 in humans can be achieved when the drug is dosed above 100 mg in adult patients.

A weight-based dosing approach was utilized in this study: infant patients were anticipated to receive a planned single dose of 4 mg/kg for Cohort 1 and 2 mg/kg for Cohort 2. The single dose (4 mg/kg for Cohort 1 and 2 mg/kg for Cohort 2, respectively) was selected to target the 5 × EC_90_ value (40 ng/mL) at 12 hours post-dose. The dose was determined using a modelling and simulation approach to predict exposure in infants through allometric scaling of adult PK parameters whilst incorporating the maturation of drug metabolizing enzyme expression. The population PK model in healthy adults was developed using single-dose data for AK0529 across the range 30‒1200 mg (Study AK0529-1001).

The SRC reviewed the blinded data at several decision points. Dose adjustment was made when necessary, as additional PK data became available throughout the study. During Part 1, dose adjustment decisions were made after completion of the first infant three patients in Cohort 1, after completion of the first nine patients in Cohort 1 (placebo, 3; AK0529, 6), after completion of the first three patients in Cohort 2, and after completion of the ninth patient in Cohort 2. During Part 2 of the study, dose adjustment decisions were made after completion of the first three patients in Cohort 3, after completion of the first three patients in Cohort 4, after completion of the ninth patient in Cohort 3, and after completion of the ninth patient in Cohort 4.

Bayesian feedback to assess the appropriateness of the selected dose level was performed using the PK data collected from the first three patients enrolled in the study. The same procedure was performed on PK data from every subsequent patient (on AK0529) enrolled in the trial. The derived exposure metrics used were C_max_, AUC_0-∞_, and C_12_.

The decision to progress to the next cohort, to continue the current cohort, and to adjust the dose level was based on the review of all available safety information, including AEs, ECGs, vital signs, clinical laboratory test results, and PK data from patients dosed. The decision by the SRC was formally documented.

**Study Schematic of Infant Patient Enrollment in Part 1 Single Dose Study**


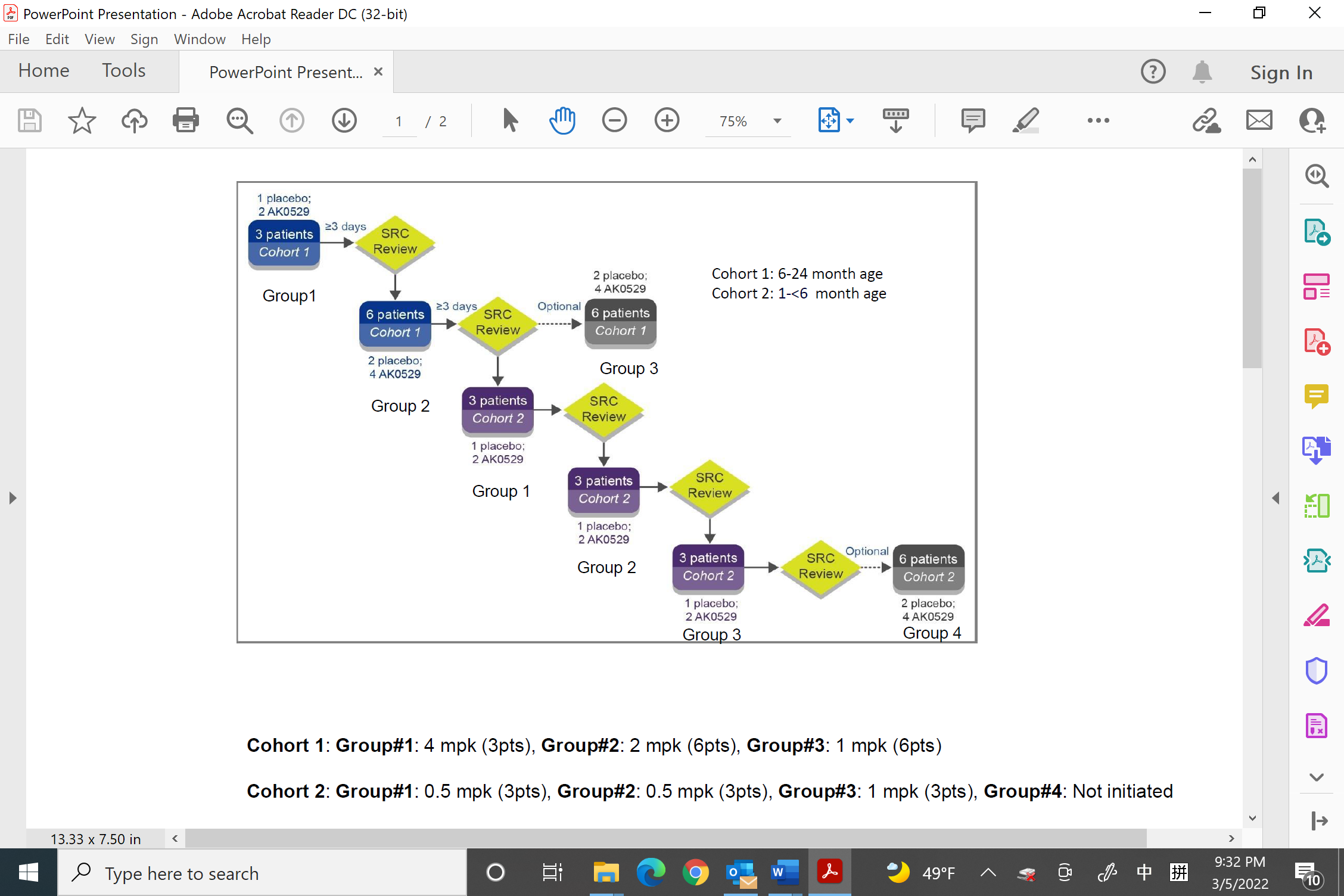


**Study Schematic of Infant Patient Enrolment in Part 2 Multiple Dose Study**


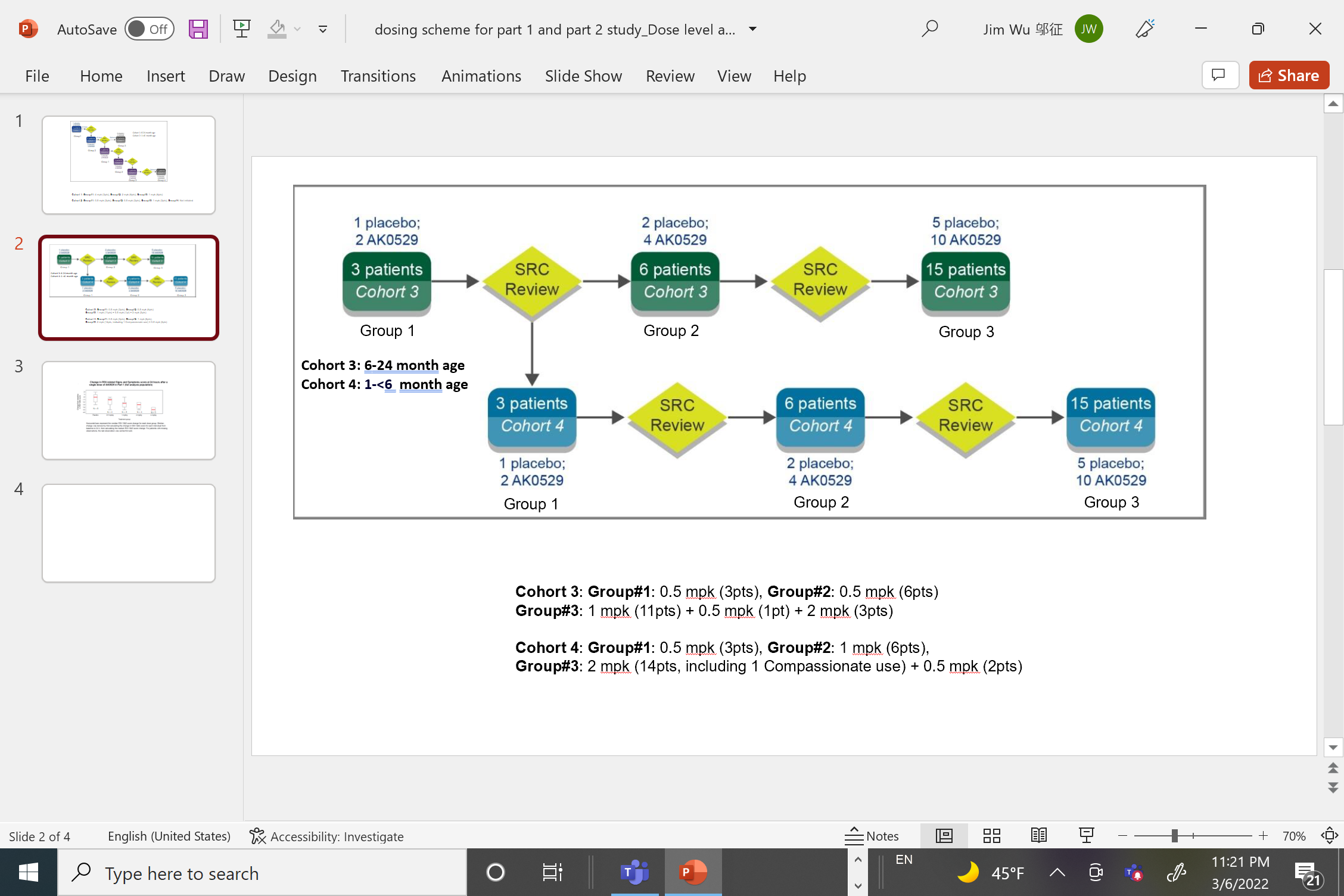


**Study Subjects and the Inclusion/Exclusion Criteria:**

Totally 73 subjects from five countries or region were enrolled in this study. The distribution of subjects in each country by age group and gender is summarized below.

| Age | Gender | Australia | Taiwan | Israel | Malaysia | Turkey |
| --- | --- | --- | --- | --- | --- | --- |
| 6 – 24 months | Female | 5 | 10 | 0 | 1 | 0 |
|  | Male | 6 | 13 | 0 | 5 | 0 |
| 1- <6 months | Female | 2 | 6 | 1 | 1 | 1 |
|  | Male | 7 | 10 | 2 | 1 | 2 |

**Inclusion Criteria**

Male or female patients of any race or ethnicity with an age adjusted for any prematurity of ≥1 month and ≤24 months.

Diagnosis of RSV infection by virological means, which may include rapid diagnostic point-of-care testing, within 96 hours preceding screening for Part 1 and 72 hours for Part 2.

Patient must weigh >3 kg at screening and be within the 10th and 90th percentiles (inclusive) for the patient’s age, based on the local child growth standards.

The parent / legal guardian of the patient must have provided written informed consent for the patient to participate.

For patients aged <12 months, an occipito-frontal head circumference within the normal range for age and gender.

**Exclusion Criteria**

The patient has taken, is currently taking or requires any restricted medications.

Patient is known to be HIV-positive (or the mother, if the potential patient is a child aged <6 months).

Participation in an investigational drug or device study within 30 days prior to the date of screening.

Requires vasopressors or inotropic support at the time of enrolment.

Concurrent gastrointestinal conditions that could, in the opinion of the investigator, prejudice absorption of the Investigational Medicinal Product (e.g. protracted vomiting, malabsorption syndrome, a history of necrotising enterocolitis with consequent short gut syndrome).

Bronchopulmonary dysplasia or chronic lung disease requiring assisted ventilation at the time of enrolment. However, any ventilation as the result of RSV illness is acceptable.

Diminished ventilatory reserve at risk for hypercapnia (e.g. pulmonary hypoplasia, sequestration syndromes, cystadenomatoid malformation, a history of surgery for diaphragmatic hernia).

Left to right shunt meriting corrective therapy.

Renal failure including renal anomalies likely to be associated with renal insufficiency (e.g. clinical conditions of renal dysplasia, polycystic renal disease, renal agenesis).

Clinical evidence of hepatic decompensation (e.g. hepatic disorder with associated coagulopathy or associated encephalopathy).

Cerebral palsy with microcephaly, chronic or persistent feeding difficulties or seizures.

Symptomatic because of inborn errors of metabolism (e.g. mitochondrial disorders, disorders of carbohydrate metabolism, glycogen storage disorders).

Congenital or acquired immunodeficiency (e.g. congenital agammaglobulinaemia, common variable immunodeficiency, immunosuppressive therapy other than glucocorticoid or monteleukast therapy forming part of care directed by the treating physician).

For Part 2 of this study, children with a history of having received palivizumab or any other monoclonal agent directed against RSV in the preceding 120 days. This exclusion criterion does not apply to Part 1.

Evidence of active or uncontrolled respiratory, cardiac, hepatic, central nervous system or renal disease unrelated to RSV infection at baseline or any other medical condition that in the opinion of the investigator renders the patient unsuitable for enrolment.

A history of epilepsy or seizures including febrile seizures.

Allergy to test medication or constituents.

Weight less than 10th percentile or greater than 90th percentile for age and gender adjusted for any prematurity.

The patient’s parent or legally acceptable representative is an employee of the investigator or the study centre, with direct involvement in the proposed study or other studies under the direction of that investigator of the study centre, or any family members of the employees or the investigator.

Failure to satisfy the investigator of fitness to participate for any other reason.

1. **Change of Wang Respiratory Score at 24 hours after a single dose of drug in Part 1 Study**


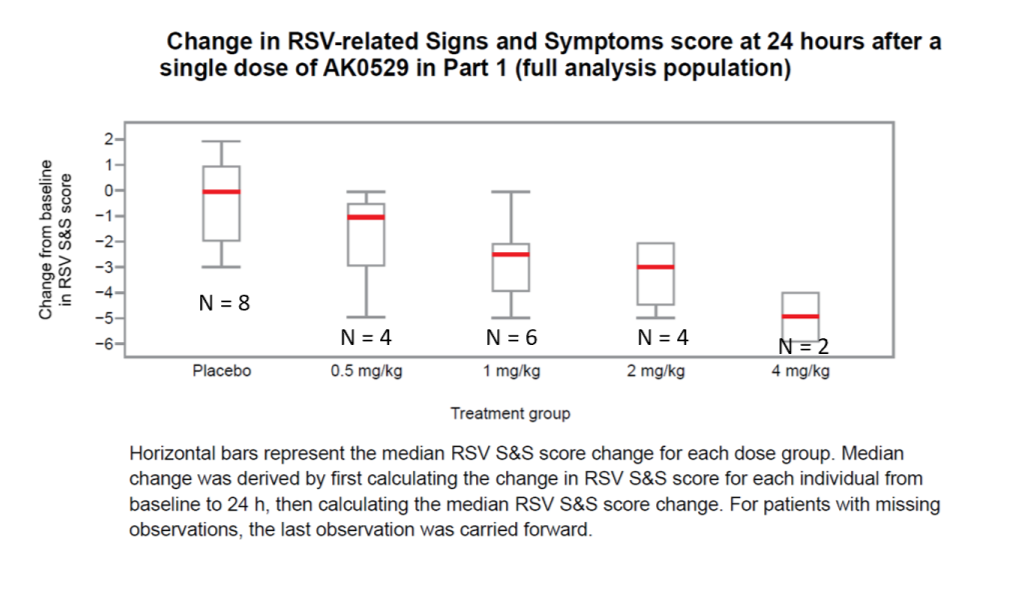


1. **Summary of TEAEs by Preferred MedDRA terms (PT) and System Organ Class (SOC) in Phase 2 Study**

Overall, 49.0% (24/49) of patients in the AK0529 group and 41.7% (10/24) in the placebo group reported grade ≥1 TEAEs. Two of the 49 (4.1%) patients in the AK0529 group and none in the placebo group experienced a serious TEAE, none of which were related to drug treatment. Grade ≥3 TEAEs were experienced by similar percentages of patients in the AK0529 and placebo groups (4.1% [2/49] and 4.2% [1/24], respectively). No withdrawals or deaths because of TEAEs were reported in the study.

| **System Organ Class Preferred Term** | **AK0529 (N=49) n (%)** | **Placebo (N=24) n (%)** | **Total (N=73) n (%)** |
| --- | --- | --- | --- |
| Patients with any TEAE | 24 (49.0%) | 10 (41.7%) | 34 (46.6%) |
| Blood and lymphatic system disorders | 3 (6.1%) | 0 | 3 (4.1%) |
| Anemia | 1 (2.0%) | 0 | 1 (1.4%) |
| Leukocytosis | 1 (2.0%) | 0 | 1 (1.4%) |
| Thrombocytosis | 1 (2.0%) | 0 | 1 (1.4%) |
| Eye disorders | 1 (2.0%) | 1 (4.2%) | 2 (2.7%) |
| Erythema of eyelid | 1 (2.0%) | 0 | 1 (1.4%) |
| Eye pruritus | 0 | 1 (4.2%) | 1 (1.4%) |
| Gastrointestinal disorders | 5 (10.2%) | 4 (16.7%) | 9 (12.3%) |
| Diarrhea | 2 (4.1%) | 1 (4.2%) | 3 (4.1%) |
| Constipation | 0 | 1 (4.2%) | 1 (1.4%) |
| Flatulence | 1 (2.0%) | 0 | 1 (1.4%) |
| Gastrointestinal inflammation | 1 (2.0%) | 0 | 1 (1.4%) |
| Hematochezia | 0 | 1 (4.2%) | 1 (1.4%) |
| Impaired gastric emptying | 0 | 1 (4.2%) | 1 (1.4%) |
| Vomiting | 1 (2.0%) | 0 | 1 (1.4%) |
| General disorders and administration site conditions | 1 (2.0%) | 3 (12.5%) | 4 (5.5%) |
| Pyrexia | 1 (2.0%) | 3 (12.5%) | 4 (5.5%) |
| Hepatobiliary disorders | 2 (4.1%) | 0 | 2 (2.7%) |
| Hepatitis | 2 (4.1%) | 0 | 2 (2.7%) |
| Infections and infestations | 5 (10.2%) | 2 (8.3%) | 7 (9.6%) |
| Otitis media acute | 2 (4.1%) | 1 (4.2%) | 3 (4.1%) |
| Acute tonsillitis | 1 (2.0%) | 0 | 1 (1.4%) |
| Bacterial infection | 0 | 1 (4.2%) | 1 (1.4%) |
| Candida infection | 1 (2.0%) | 0 | 1 (1.4%) |
| Conjunctivitis | 1 (2.0%) | 0 | 1 (1.4%) |
| Oral candidiasis | 1 (2.0%) | 0 | 1 (1.4%) |
| Injury, poisoning and procedural complications | 1 (2.0%) | 0 | 1 (1.4%) |
| Injection site extravasation | 1 (2.0%) | 0 | 1 (1.4%) |
| Investigations | 6 (12.2%) | 2 (8.3%) | 8 (11.0%) |
| Aspartate aminotransferase increased | 3 (6.1%) | 0 | 3 (4.1%) |
| Transaminases increased | 2 (4.1%) | 1 (4.2%) | 3 (4.1%) |
| Rotavirus test positive | 0 | 1 (4.2%) | 1 (1.4%) |
| Urine ketone body present | 1 (2.0%) | 0 | 1 (1.4%) |
| Metabolism and nutrition disorders | 4 (8.2%) | 0 | 4 (5.5%) |
| Hyperkalemia | 2 (4.1%) | 0 | 2 (2.7%) |
| Hyperphosphatemia | 1 (2.0%) | 0 | 1 (1.4%) |
| Metabolic acidosis | 1 (2.0%) | 0 | 1 (1.4%) |
| Respiratory, thoracic and mediastinal disorders | 6 (12.2%) | 3 (12.5%) | 9 (12.3%) |
| Pneumonia | 3 (6.1%) | 1 (4.2%) | 4 (5.5%) |
| Epistaxis | 2 (4.1%) | 1 (4.2%) | 3 (4.1%) |
| Nasopharyngitis | 1 (2.0%) | 1 (4.2%) | 2 (2.7%) |
| Upper respiratory tract infection | 1 (2.0%) | 0 | 1 (1.4%) |
| Skin and subcutaneous tissue disorders | 6 (12.2%) | 1 (4.2%) | 7 (9.6%) |
| Dermatitis diaper | 4 (8.2%) | 0 | 4 (5.5%) |
| Rash | 1 (2.0%) | 1 (4.2%) | 2 (2.7%) |
| Dermatitis | 1 (2.0%) | 0 | 1 (1.4%) |

1. **Analysis of ALT and AST Changes during Treatment**

As hospitalized infants with RSV infection commonly have elevated AST and ALT levels [Do et al., 2016; Eisenhut et al., 2004] and early elevation of AST levels may be predictive of disease severity and risk of death on pediatric intensive care unit (PICU) admission [Thorburn et al., 2018], ALT and AST changes in the study were further assessed.

Seven patients (11.9% [7/59]) had elevated ALT baseline values above the upper limit of the normal range. Among these, five patients received AK0529 and two patients received placebo. Additionally, AST values were outside the upper limit of normal range at baseline for 23 patients (37.1% [23/62]), with 17 of these patients receiving AK0529 and six receiving placebo.

ALT and AST shifts from baseline were evaluated for all patients with available data according to seven shift categories. No statistically significant differences between the AK0529 and placebo groups were observed with respect to shifts in ALT or AST levels.
